# Diet-associated variation in the midgut bacterial communities of *Loxostege sticticalis* larvae fed botanically distinct host plants

**DOI:** 10.3389/finsc.2026.1949678

**Published:** 2026-09-28

**Authors:** Dong Li, Jianyu Wang, Dandan Zhang, Weichan Cui, Haijun Xiao

**Affiliations:** School of Grassland Science, Beijing Forestry University, Beijing, China

**Keywords:** gut bacteria, diet -microbiota interactions, microbial communities, polyphagous pest, predicted microbial functions

## Abstract

**Introduction:**

Gut microbiota can influence insect physiology and ecological interactions, but how botanically distinct host plants affect gut bacterial communities in polyphagous insects remains poorly understood.

**Methods:**

We investigated the midgut bacterial communities of *Loxostege sticticalis* larvae fed three host plants from different genera or an artificial diet using 16S rRNA gene sequencing. Bacterial diversity, community composition, differential taxa, and predicted functional profiles were compared among dietary treatments.

**Results:**

Dietary treatments were associated with differences in gut bacterial community structure. The MS group exhibited higher bacterial richness and diversity, whereas the CA group showed a relatively simplified bacterial community. Bray–Curtis-based PCoA and PERMANOVA indicated significant overall variation in community composition among dietary treatments, although exact pairwise PERMANOVA tests were not significant. Bacillota and Pseudomonadota were dominant across treatments, while the MS group showed increased representation of Actinomycetota-associated taxa. LEfSe identified Actinomycetota-related taxa as characteristic features of the MS group and Bacillota-related taxa as features of the AD group. PICRUSt2 suggested differences in predicted functional potential among dietary treatments, including pathways related to amino acid metabolism, metabolism of cofactors and vitamins, and xenobiotics biodegradation and metabolism.

**Discussion:**

The results demonstrate diet-associated variation in the gut bacterial communities of *L. sticticalis* and provide a descriptive basis for further investigation of diet–microbiota interactions. Because the study included only three pooled biological replicates per treatment and did not characterize plant-associated microbiota or plant chemistry, the observed associations and predicted functions should not be interpreted as evidence of specific microbial mechanisms or host adaptation.

## 1 Introduction

The gut microbiota could influence multiple aspects of insect biology. In herbivorous insects, gut-associated microorganisms may contribute to food digestion, nutrient acquisition, immune regulation, pathogen resistance, and the metabolism or tolerance of plant defensive compounds (1–3). However, insect-associated microbial communities are not fixed. Their diversity and composition may vary with host phylogeny, diet, developmental stage, habitat, and other environmental conditions (1, 4). Moreover, the functional importance of gut microorganisms differs considerably among insect lineages. Some insects maintain stable symbiotic bacteria that provide essential nutrients or contribute to development and defense (1, 3), whereas many caterpillars harbor relatively sparse, environmentally acquired communities and may lack a persistent resident gut microbiome (5, 6).

Among the factors influencing gut microbiota, diet is one of the most direct factors shaping insect gut microbial communities because ingested food alters gut physicochemical conditions and supplies nutrients, plant secondary metabolites, and food-associated microorganisms (2, 4, 5, 7). Recent research has increasingly recognized that host plants may influence insect physiology and ecological performance indirectly through their effects on gut bacterial communities and microorganisms from plant surfaces and internal tissues may also persist temporarily after ingestion (5, 8, 9). Consequently, host plants could shape gut bacterial communities through both selective conditions within the gut and passive acquisition of food-associated bacteria. For example, termite-associated bacteria play indispensable roles in lignocellulose degradation and nitrogen metabolism, enabling termites to efficiently exploit woody diets (10), in conifer-feeding weevils whose bacteria metabolize diterpenes (11), and in polyphagous lepidopteran pests such as *Tuta absoluta*, *Spodoptera frugiperda*, and *Spodoptera exigua* (9, 12–15). In contrast, many Lepidoptera harbor relatively simple and transient gut bacterial communities that are strongly influenced by diet and environmental exposure rather than stable host-specific symbioses (6, 16). Controlled work in *S. frugiperda* further indicates that microbial source, plant genotype, and their interaction can all affect gut community structure (14). Therefore, a key step in understanding whether the gut microbiota is related to plant utilization and dietary adaptation in polyphagous insect hosts is to study the correlation between gut microbiota and host plant changes.

However, despite some progress in existing research, several key questions remain unresolved. Most current studies have compared gut microbial communities among closely related crop species or within a relatively narrow range of host plants, making it difficult to distinguish general host plant effects from species-specific responses. It remains unclear whether different genera of host plants could influence and shape the gut bacterial community of insects, rather than simply representing a transient input of food-derived microorganisms (5, 17). This gap is particularly evident for *Loxostege sticticalis*, an economically important polyphagous pest capable of utilizing taxonomically diverse host plants (18, 19), for which systematic comparisons across botanically distinct hosts are still lacking.

In this study, we used 16S rRNA gene amplicon sequencing to compare the gut bacterial communities of *L sticticalis* larvae reared on three different genera of host plants and an artificial diet. We analyzed bacterial diversity, community composition, and predicted functional profiles across dietary treatments. The aim of this study was to evaluate whether different genera of host plants were associated with consistent differences in larval gut bacterial communities, including bacterial diversity, taxonomic composition, and predicted functions. Our findings provide a foundation for understanding diet-associated gut microbiota variation in *L. sticticalis* and for future studies of diet -microbiota interactions.

## 2 Materials and methods

### 2.1 Test insects and rearing conditions

A laboratory colony of *L. sticticalis* was established from adult insects collected in Hohhot, Inner Mongolia Autonomous Region, China (110°31′–111°53′ E, 40°47′–41°53′ N). After oviposition and egg hatching, the larvae were maintained on fresh *Chenopodium album* leaves. Rearing was conducted in an incubator at 21 ± 1 °C, under a 16 h light:8 h dark photoperiod, and 70–80% relative humidity. Adult insects were supplied with 10% honey water solution (w/v) as food and maintained under the same environmental conditions.

### 2.2 Diet treatments

The four diet treatments consisted of three host plants, *C. album* (genus *Chenopodium*), *Medicago sativa* (alfalfa, genus *Medicago*), *Beta vulgaris* (sugar beet, genus *Beta*) and an artificial diet.

Fresh, visibly healthy, and insect-free *C. album* plants were collected at random from the experimental field. Alfalfa and sugar beet were cultivated under hydroponic conditions. Seeds of alfalfa and sugar beet were first pre-treated by soaking in water for 24 h, drained and evenly spread on filter paper in Petri dishes; the moisture was maintained by daily misting to promote germination. After germination, seedlings were transferred to containers with Hoagland nutrient solution for continued hydroponic cultivation. Leaves were harvested when the plants had developed four to six young leaves and were immediately provided to the larvae. For all three plant diets, only young, intact leaves without visible chlorosis, disease lesions, herbivore damage, surface insects, or adhering soil were used, and food was supplied immediately after collection. The artificial diet for *L. sticticalis* larvae was prepared according to the previous study (20). Each diet was treated as a composite exposure that includes both plant traits and potential food-associated microbial inputs.

### 2.3 Experimental design and sample collection

Larvae were assigned to four diet groups: CA (*C. album*), MS (*M. sativa*), BV (*B. vulgaris*), and AD (artificial diet). After two generations of laboratory maintenance on *C. album*, eggs from the same parental cohort were collected, and newly hatched larvae were randomly assigned to the four diet treatments and maintained at 21 ± 1 °C, 70–80% relative humidity, and a 16 h light:8 h dark photoperiod. Food (artificial diet or fresh leaves) was provided to the 1st-3rd instar larvae every 2 days. When they reach the 4th and 5th instars, provide food daily, ensuring that the food is sufficient and fresh. At the 5th instar, larvae were considered externally healthy when they showed normal movement upon gentle stimulation via toothpick, an intact cuticle, no visible discoloration, fungal growth, or injury, and a body size consistent with other individuals of the same instar. Larvae meeting these criteria were randomly selected for sampling.

Samples were collected according to the method previously reported by Wang et al. (21). To reduce the possibility of epidermal microbial residues on the diversity of the intestinal bacterial community, the collected larvae were transferred into an ice box for 15 min chilling, immersed in 75% ethanol solution for 2–3 min and washed with sterile water for 15–30 s; rinsing was repeated when visible debris remained. Excess water was removed with sterile filter paper, and larvae were dissected under a microscope (LEICA M250C), each larva was held with sterile forceps, a small transverse opening was made near the posterior end, and the intact alimentary canal was gently withdrawn without compressing or rupturing the gut. The foregut and hindgut were separated at their junctions with the midgut, and the isolated midgut was transferred to a sterile 1.5-mL tube, immediately frozen in liquid nitrogen, and stored at −80 °C until DNA extraction. For each treatment, three pooled biological replicates were prepared, each consisting of midguts from 20 larvae of similar size and was treated as the experimental unit for downstream statistical analyses.

### 2.4 DNA extraction and 16S rRNA sequencing

DNA was extracted from each pooled gut sample using a cetyltrimethylammonium bromide (CTAB) method. The DNA concentration and purity were assessed using a NanoDrop 2000 spectrophotometer (Thermo Scientific, DE, USA), with samples exhibiting A260/A280 ratios between 1.8 and 2.2 considered acceptable for downstream applications. The DNA integrity was verified using 1% agarose gel electrophoresis. The V3–V4 hypervariable region of the bacterial 16S rRNA gene was amplified using the universal primers 341F (5′-CCTAYGGGRBGCASCAG-3′) and 806R (5′-GGACTACNNGGGTATCTAAT-3′), based on previous reports (22). Each 30 μL PCR reaction contained 15 μL of 2× Phusion Master Mix, 1 μL of each primer, 10 μL of template DNA, and 3 μL of nuclease-free water. The PCR cycling conditions were as follows: initial denaturation at 98 °C for 1 min; 30 cycles of 98 °C for 10 s, 50 °C for 30 s, and 72 °C for 30 s; and a final extension at 72 °C for 5 min. An extraction blank containing no biological material was processed in parallel with the gut samples throughout the DNA extraction procedure and was subsequently subjected to PCR amplification. In addition, a no-template PCR negative control containing sterile water instead of template DNA was included in each amplification. Neither the extraction blank nor the PCR negative control produced a visible amplification band on agarose gels. PCR products were separated on a 2% agarose gel, which provides suitable resolution for the approximately 460-bp V3–V4 amplicons and facilitates their discrimination from shorter nonspecific products. The target bands were excised and purified using a Universal DNA Purification & Recovery Kit (TianGen, China) according to the manufacturer’s instructions.

Libraries were prepared using the NEBNext^®^ Ultra DNA Library Prep Kit. Library quality was evaluated using an Agilent 5400 system, and library concentrations were determined by qPCR. The libraries were sequenced on an Illumina platform to generate 2 × 250-bp paired-end reads.

### 2.5 Bioinformatics and statistical analysis

Raw sequencing reads (FASTQ files) were imported into QIIME 2 for processing. DADA2 plugin was used for quality filtering, trimming, denoising, read merging, and chimera removal to generate amplicon sequence variant (ASV) tables (23, 24). Representative ASVs were assigned taxonomy against SILVA release 138 with the q2-feature-classifier plugin (25). Sequences identified as likely mitochondrial or chloroplast contaminants were removed. Rarefaction curves were generated to evaluate sampling coverage, and samples were rarefied to a common depth corresponding to the minimum retained sequencing depth before diversity analyses.

Alpha diversity of the midgut bacterial communities was evaluated using the Chao1 richness estimator, Shannon diversity index, Simpson diversity index, and Pielou’s evenness index. Differences in alpha-diversity indices among the four diet groups were analyzed using one-way analysis of variance (ANOVA) in SPSS Statistics 27.0 (IBM, Armonk, NY, USA). When the overall ANOVA was significant *(P* < 0.05), Tukey’s honestly significant difference (HSD) test was used for *post hoc* pairwise comparisons. Statistical significance was set at *P* < 0.05.

Beta diversity was visualized using principal coordinate analysis based on Bray–Curtis dissimilarities. Homogeneity of multivariate dispersion among dietary treatments was assessed using PERMDISP based on the same Bray–Curtis dissimilarity matrix and distances to group spatial medians were calculated using the betadisper procedure, and significance was evaluated by permutation testing, Overall differences in community composition were tested using PERMANOVA implemented with the adonis2 function in the vegan package for R(Vienna, Austria), with 999 permutations (26, 27). Because each pairwise comparison contained only six samples (three per group), pairwise PERMANOVA was additionally evaluated using all possible group-label allocations to obtain exact permutation *P* values. Taxonomic composition at various ranks was summarized and visualized as relative abundance bar plots. Differences in specific bacterial taxa between groups were identified using Linear Discriminant Analysis Effect Size (LEfSe) on the Wekemo Bioincloud platform (Wekemo Tech Group Co., Ltd., Shenzhen, China), with an LDA score > 4 (28). PICRUSt2 on the same platform was used to infer KEGG pathway profiles from 16S rRNA data (29). These profiles are predictions rather than measurements of genes, transcripts, metabolites, or enzyme activities.

## 3 Results

### 3.1 Sequencing and taxonomic overview

Across the 12 pooled midgut samples of *L. sticticalis* larvae, 1,216,636 raw reads targeting the bacterial 16S rRNA gene were obtained. After quality filtering and chimera removal, 985,571 high-quality reads were retained. Following rarefaction to the common minimum sequencing depth, 1,738 unique ASVs were retained across the complete dataset (**Table 1**).

**Table 1:** Taxonomic coverage of gut bacterial sequences in *Loxostege sticticalis* larvae feeding on different hosts (number of taxa at each rank and total ASV count per sample).

Sample	Phylum	Class	Order	Family	Genus	Species	ASV counts
CA1	9	10	34	38	39	26	72
CA2	8	10	18	22	20	9	54
CA3	11	14	32	46	55	37	87
MS1	21	27	77	127	293	313	819
MS2	18	26	73	114	238	245	556
MS3	22	30	82	124	282	278	680
BV1	18	27	51	73	96	58	156
BV2	6	7	11	12	15	8	33
BV3	7	8	16	23	26	17	50
AD1	4	5	12	14	20	8	36
AD2	3	4	9	10	13	6	32
AD3	5	5	9	11	13	7	33
**Dataset-wide unique**	**28**	**45**	**124**	**195**	**470**	**513**	**1738**

CA – larvae feeding on *C. album*; MS – on *M. sativa*; BV – on *B. vulgaris*; AD – on artificial diet. The final row reports the number of ASVs detected across the complete dataset after pooling and de-duplication across samples; it is therefore not expected to equal the arithmetic sum of the per-sample counts, because the same ASV can occur in multiple samples. Bold values represent the dataset-wide numbers of unique taxa detected at each taxonomic rank and the total number of unique ASVs after pooling and de-duplicating all samples. These values are not arithmetic sums of the corresponding per-sample counts because the same taxon or ASV may occur in multiple samples.

Rarefaction curves were used to evaluate sampling coverage for all samples. As sequencing depth increased, the curves approached asymptotes (**Figure 1**), suggesting that the retained sequencing effort captured most of the ASV richness detectable under the applied pipeline. Because all samples were rarefied to a common sequencing depth before diversity analysis, differences in sequencing effort were minimized when comparing bacterial richness among dietary treatments.

**Figure 1:**
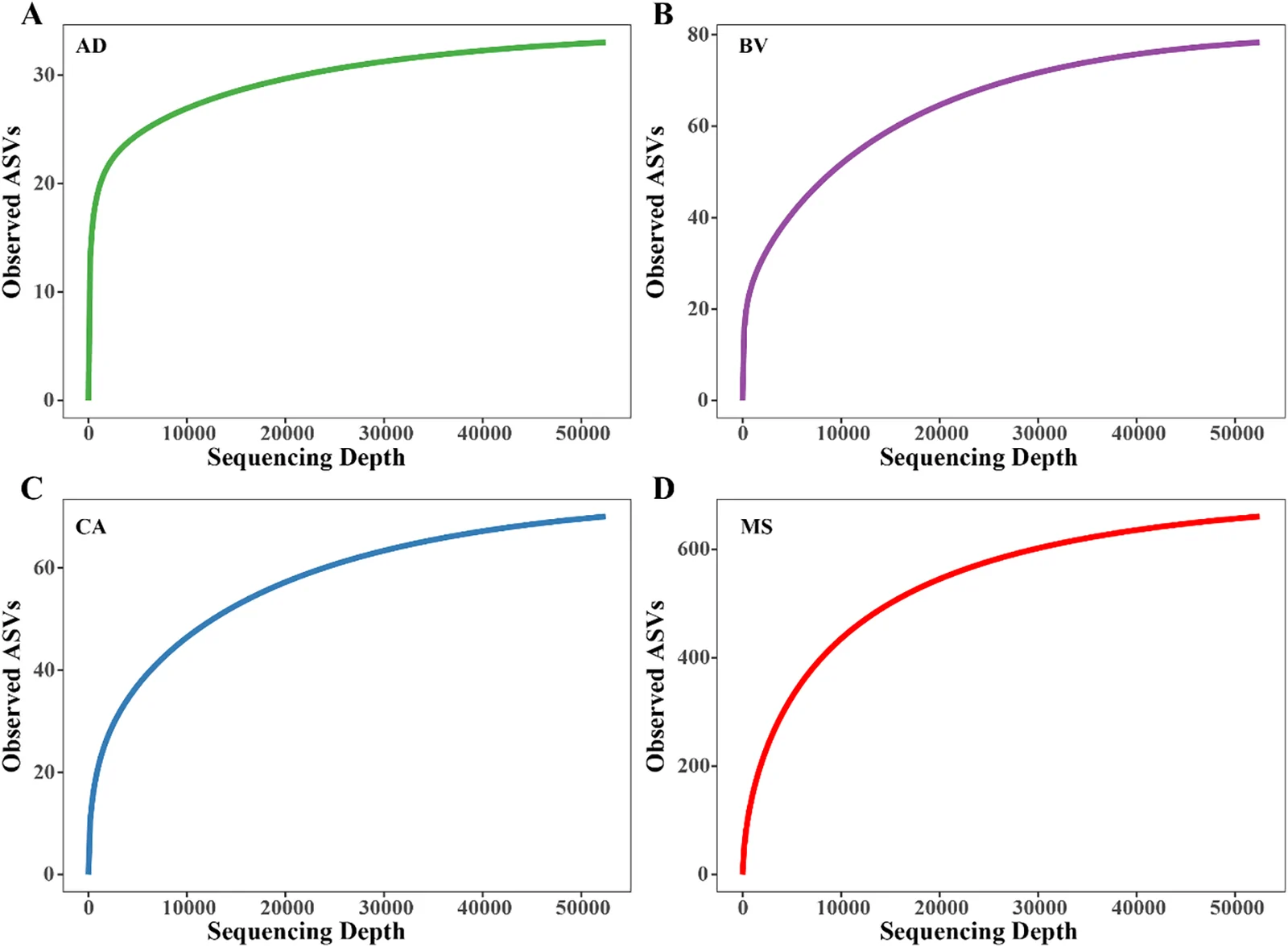
Rarefaction curves of observed ASVs in the midgut bacterial communities of *L. sticticalis*. **(A–D)** Rarefaction curves based on the ASVs for groups **(A)** AD, **(B)** BV, **(C)** CA, and **(D)** MS. The curves depict the relationship between sequencing depth (number of reads) and species richness.

### 3.2 Diversity of gut bacterial communities in *L. sticticalis* larvae

Alpha-diversity metrics differed among dietary treatments (one-way ANOVA followed by Tukey’s HSD test, *P* < 0.05; **Figure 2A**). Through the analysis of the diversity index of intestinal bacteria, we found that the MS group had significantly higher Chao1 richness, and Shannon diversity than the AD, BV, and CA groups. For evenness and Simpson diversity, MS and AD shared the same significance and did not differ significantly, CA was lower than MS and AD, whereas BV did not differ from either group. Although the median Chao1 value was lowest in AD and the median Shannon value was lowest in CA, AD, BV, and CA shared the same Tukey significance group for both metrics. Therefore, treatment differences were interpreted according to the *post hoc* significance groups rather than the numerical ranking of median values.

**Figure 2:**
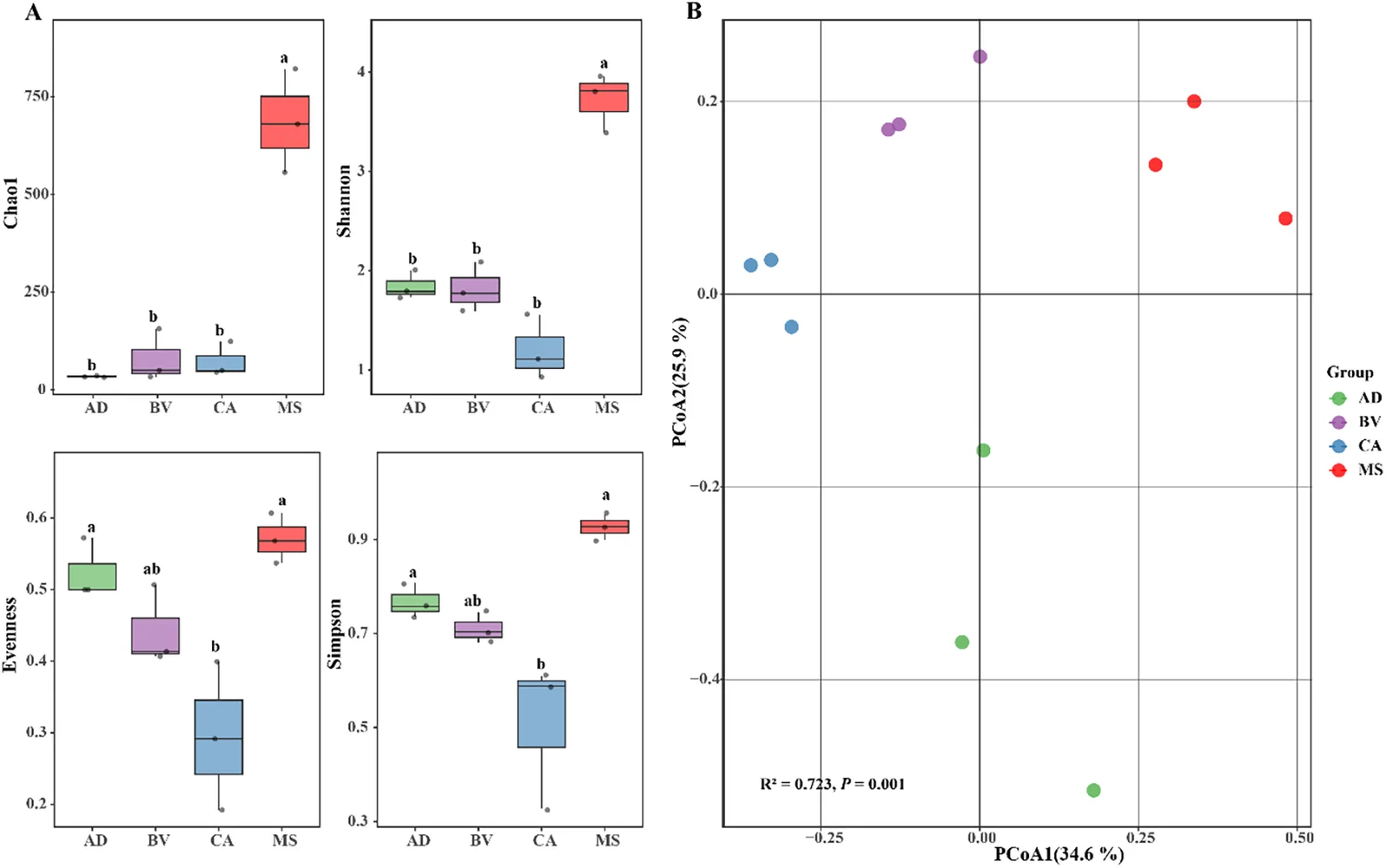
Alpha and beta diversity of midgut bacterial communities under different dietary treatments. **(A)** Boxplots representing alpha diversity indices: Chao1, Shannon, Evenness, and Simpson indices. Different lowercase letters (a, b) above the boxes indicate statistically significant differences between groups (one-way ANOVA with Tukey’s *post hoc* test, *P* < 0.05). The horizontal line represents the median, and whiskers indicate the range. **(B)** Bray–Curtis PCoA of bacterial community composition. PERMDISP detected no significant heterogeneity of multivariate dispersion among treatments (F_3,8_ = 0.136, *P* = 0.94), whereas the overall PERMANOVA detected a significant diet-associated difference in community composition (R² = 0.723, *P* = 0.001). None of the pairwise PERMANOVA comparisons was significant.

We compared the overall bacterial community composition among the four dietary treatments. A principal coordinate analysis (PCoA) based on Bray–Curtis dissimilarities was shown in **Figure 2B**. The first and second principal coordinates explained 34.6% and 25.9% of the total variation, respectively. The ordination showed different distributions of samples among treatments, and these two dimensional patterns were interpreted descriptively. PERMDISP based on the full Bray–Curtis distance matrix detected no significant heterogeneity in multivariate dispersion among the four treatments (F_3,8_ = 0.136, *P* = 0.94). Mean distances to the group spatial medians were 0.167 for CA, 0.223 for AD, 0.246 for BV, and 0.215 for MS.

PERMANOVA was used to test whether overall bacterial community composition differed among dietary treatments. The overall detected a significant diet-associated difference (R² = 0.723, *P* = 0.001, **Table 2**). However, exact pairwise permutation tests yielded *P* = 0.100 for each of the six treatment contrasts. Therefore, the statistical evidence supports an overall treatment-associated difference in multivariate community composition but does not identify a significant difference attributable to any specific pair of dietary treatments.

**Table 2:** PERMANOVA results comparing gut bacterial community composition among dietary treatments based on Bray–Curtis dissimilarities.

Comparison	Sample size	Permutations	*P*-value
CA vs. MS	6	999	0.100
CA vs. AD	6	999	0.100
CA vs. BV	6	999	0.100
MS vs. AD	6	999	0.100
MS vs. BV	6	999	0.100
AD vs. BV	6	999	0.100
Overall (all groups)	12	999	0.001

The overall test used 999 permutations; pairwise tests used exact permutations because each contrast contained three samples per group, For each pairwise contrast (3 vs. 3 samples), all possible group-label allocations were evaluated. all exact pairwise *P* values were 0.100. *P* > 0.05 indicates that the corresponding pairwise difference was not statistically significant.

### 3.3 Gut bacterial community composition in larvae of *L. sticticalis*

To characterize the taxonomic composition of gut bacterial communities among the four dietary treatments (AD, BV, CA, and MS), the relative abundances of bacterial taxa were compared at the phylum and genus levels (**Figure 3**). At the phylum level, Bacillota and Pseudomonadota were the dominant bacterial groups across all dietary treatments, accounting for the majority of the detected sequences (**Figure 3A**). However, their relative proportions varied among diets. The AD and BV groups showed similar phylum-level profiles, with Bacillota and Pseudomonadota representing the predominant bacterial phyla. In contrast, the CA group exhibited a strong dominance of Bacillota, whereas Pseudomonadota showed a lower relative abundance. The MS group displayed a more diverse phylum-level composition, characterized by increased relative abundances of Actinomycetota and other less dominant bacterial phyla compared with the other treatments.

**Figure 3:**
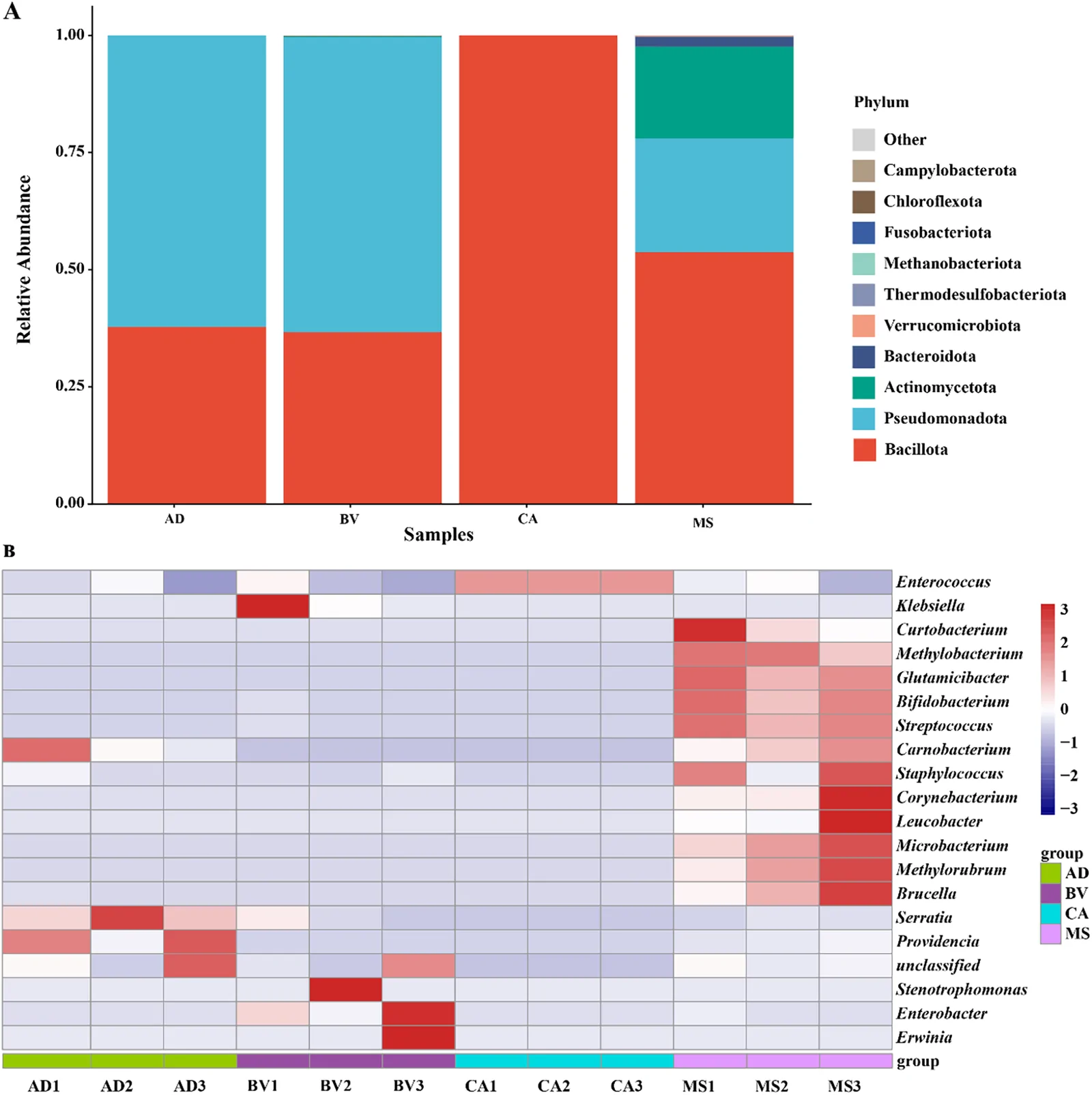
Taxonomic composition of *L. sticticalis* larval gut bacteria under different host diets: **(A)** phylum-level relative abundance and **(B)** genus-level relative abundance.

At the genus level, the heatmap revealed distinct distribution patterns of bacterial taxa among dietary groups (**Figure 3B**). The MS group showed relatively higher abundances of several genera, including *Curtobacterium*, *Methylobacterium*, *Glutamicibacter*, *Bifidobacterium*, *Streptococcus*, and *Corynebacterium*. The AD group exhibited higher relative abundances of several genera, including *Carnobacterium*, *Serratia*, and *Providencia*. In the BV group, genera such as *Klebsiella*, *Stenotrophomonas*, and *Enterobacter* showed relatively higher abundances in specific replicates. Compared with the other dietary groups, the CA group exhibited relatively low abundances of most genera displayed in the heatmap, with fewer dominant bacterial taxa detected.

Overall, these results demonstrate that dietary treatments were associated with differences in gut bacterial community composition. The MS group exhibited a relatively broader taxonomic profile, consistent with its higher alpha diversity observed in previous analyses, whereas the CA group showed a comparatively simplified community composition.

To identify taxa showing the strongest treatment-associated differences, LEfSe analysis was performed across the four dietary groups. Differential taxa meeting both *P* < 0.05 and LDA > 4.0 were detected only in the MS and AD groups (**Figure 4**).

**Figure 4:**
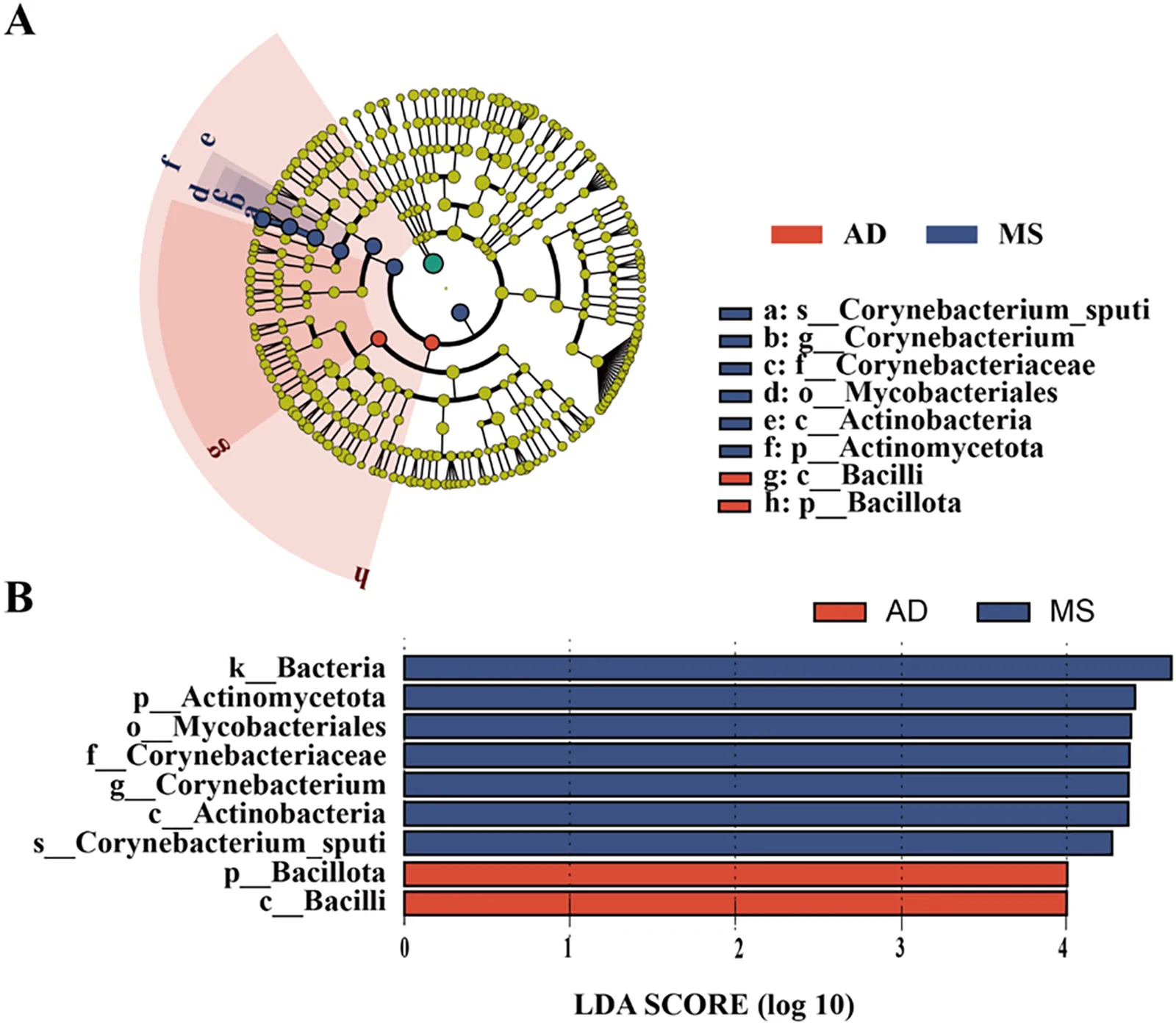
LEfSe analysis of gut bacteria in *L. sticticalis* larvae feeding on different diets. **(A)** The cladogram shows bacterial taxa (from phylum to species level) that were significantly different among diet groups based on LEfSe analysis (LDA > 4.0, *P* < 0.05). Different colors indicate the dietary groups in which the corresponding taxa were enriched **(B)** The bar chart shows the LDA scores of the differentially abundant taxa identified by LEfSe, with higher scores indicating greater differences in relative abundance among groups.

In the MS group, the LEfSe features mainly belonged to Actinomycetota, including Actinomycetota at the phylum level, Actinobacteria at the class level, Mycobacteriale*s* at the order level, Corynebacteriaceae at the family level, *Corynebacterium* at the genus level, and *Corynebacterium sputi* at the species level. In the AD group, the detected differential features mainly belonged to Bacillota and Bacilli. The LDA scores showed that all identified differential taxa exceeded the selected threshold (LDA > 4.0). No feature in the CA or BV group met both the statistical significance and effect-size thresholds (*P* < 0.05 and LDA > 4.0) under the present dataset. This result indicates only that no taxa satisfied both LEfSe criteria in these groups and should not be interpreted as evidence that characteristic or biologically relevant bacterial taxa were absent from CA or BV fed larvae.

### 3.4 Predicted functional profiles of the gut microbiota

To investigate the potential functional profiles of gut bacterial communities under different dietary treatments, PICRUSt2 was used to predict KEGG pathway abundances based on 16S rRNA gene sequencing data. The predicted functions were summarized at KEGG Level 1 and Level 2 (**Figure 5**). At KEGG Level 1, metabolism was the most dominant predicted functional category in all four diet groups, accounting for the largest proportion of predicted pathway abundance, followed by genetic information processing, cellular processes, human diseases, environmental information processing, and organismal systems (**Figure 5A**). The overall distribution of predicted functional categories was generally similar among the AD, BV, CA, and MS groups.

**Figure 5:**
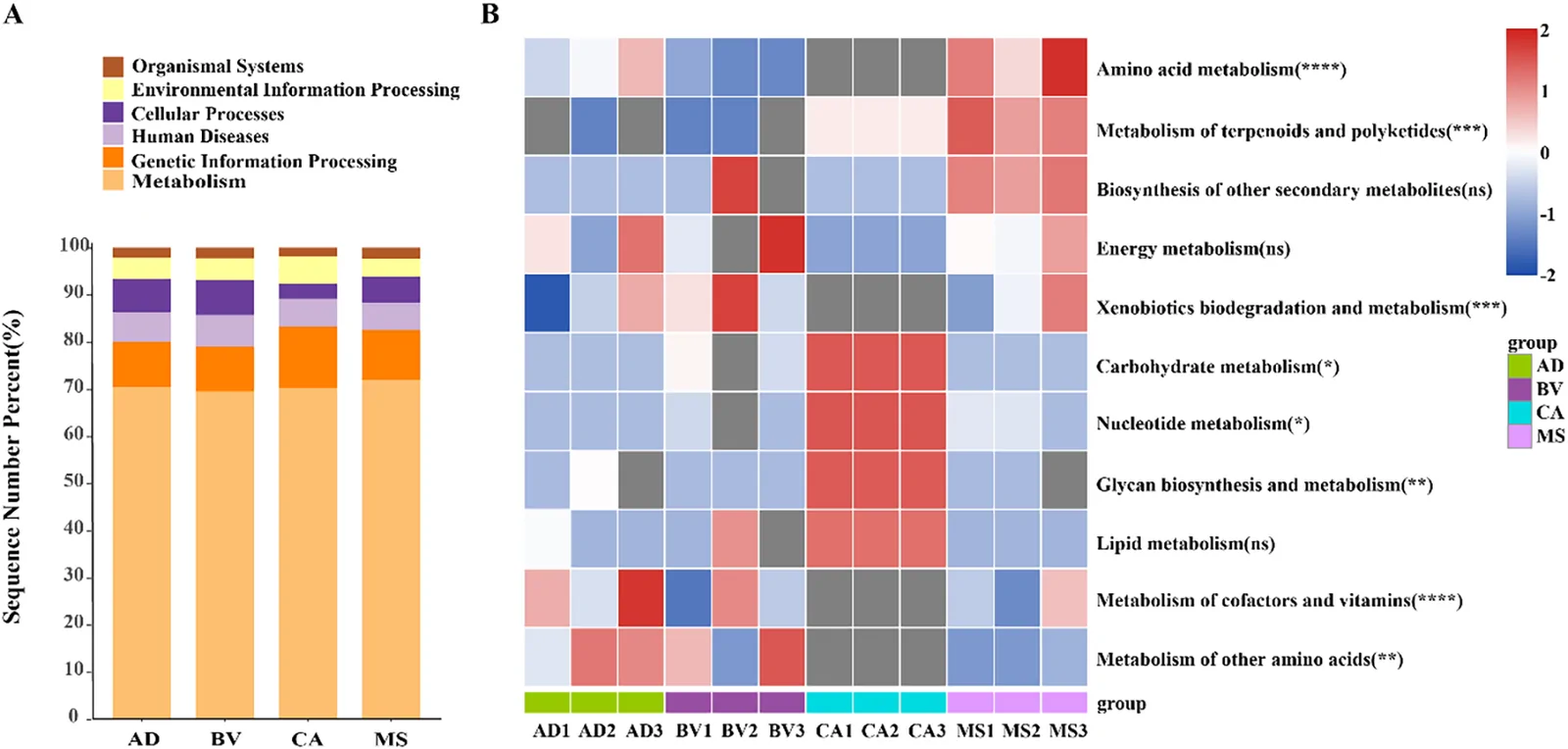
Functional prediction analysis of bacterial communities in *L. sticticalis* larvae fed different diets based on PICRUSt2. **(A)** Stacked bar chart showing the relative abundances of predicted KEGG Level 1 functional categories. **(B)** Clustering heatmap illustrating the relative abundances of major differential metabolic pathways at KEGG Level 2. Asterisks following the pathway names denote the level of statistical significance for differences in relative abundance among the groups (one-way ANOVA ns, not significant; *P < 0.05; **P < 0.01; ***P < 0.001; ****P < 0.0001).

At KEGG Level 2, the categories annotated as significantly different among groups were amino acid metabolism *(P* < 0.0001), metabolism of terpenoids and polyketides (*P* < 0.001), xenobiotics biodegradation and metabolism (*P* < 0.001), carbohydrate metabolism (*P* < 0.05), nucleotide metabolism (*P* < 0.05), glycan biosynthesis and metabolism (P < 0.01), metabolism of cofactors and vitamins (*P* < 0.0001), and metabolism of other amino acids (*P* < 0.01) (**Figure 5B**). The MS group showed higher predicted relative abundances of amino acid metabolism and terpenoid and polyketide metabolism categories. The CA group showed higher predicted relative abundances of carbohydrate metabolism, nucleotide metabolism, and glycan biosynthesis and metabolism categories. The AD and BV groups also displayed distinct patterns of localized high abundance in specific pathways, such as cofactor and vitamin metabolism.

## 4 Discussion

In this study, we found that dietary treatments were associated with variation in the diversity, composition, and PICRUSt2-predicted functional profiles of the midgut bacterial communities of *L. sticticalis*. Similar diet associated microbiome variation has been reported in other insects, including herbivorous Lepidoptera (4, 5, 9, 30, 31). However, most previous studies have focused on closely related host plants or specific environmental conditions. By comparing three botanically distinct host plants together with an artificial diet, our study provides a descriptive baseline for microbiome variation across contrasting food sources. Importantly, the present 16S rRNA sequencing data demonstrate associations between diet and microbiome variation rather than direct microbial effects on host-plant adaptation. Therefore, the observed bacterial taxa and predicted pathways should be considered candidates for future functional investigations.

The diversity analysis indicated that MS groups showed relatively higher Chao1 and Shannon indices compared with other groups, suggesting greater bacterial richness and diversity under this dietary condition. The higher bacterial richness and diversity observed in MS group may be associated with differences in host plant characteristics. Compared with the other diets, *M. sativa* may differ in nutritional composition, secondary metabolites, and microorganisms associated with the food source, any of which could contribute to the higher bacterial diversity observed in the MS group. In contrast, the relatively lower diversity observed in CA group may reflect potential selective effects of plant-derived compounds or differences in dietary characteristics that influence bacterial colonization and persistence.

The present study did not characterize bacterial communities associated with the surfaces or internal tissues of the host plants. Therefore, we cannot determine to what extent the observed gut bacterial differences resulted from direct acquisition of food-associated microorganisms versus selective effects imposed by dietary nutritional or chemical properties. However, previous studies indicate that these processes are not mutually exclusive. Dietary plant chemistry has been shown to influence the midgut bacterial community of lepidopteran larvae (13). Maize genotype continued to influence gut bacterial community membership and structure in axenic *S. frugiperda* larvae provided with a standardized microbial inoculum and gamma-irradiated maize leaves designed to minimize continued bacterial input from the plants (14). Diet-dependent changes in metabolically active gut bacterial communities have also been observed in *S. exigua* maintained under laboratory conditions on different artificial diet formulations (15). These studies suggest that diet can influence the gut bacterial community of lepidopteran insects, rather than simply passively acquiring microorganisms from food. Thus, the patterns observed in this study are most interpreted as diet-associated variation potentially arising from a combination of diet microbial input and dietary selection within the gut, rather than as evidence of selective colonization alone.

The Bray–Curtis analyses support an overall association between dietary treatment and bacterial community composition. PERMANOVA indicated significant variation among the four diet groups, but none of the exact pairwise tests was significant, reflecting the limited resolution of comparisons with three pooled replicates per group. Similar host-associated patterns have been reported in other polyphagous Lepidoptera, where phyllosphere inputs, plant chemistry, gut physiology, and stochastic variation can jointly influence the detected microbiota (12, 14, 15).

At the phylum level, analysis further supported the association between diet and gut bacterial community structure. Bacillota and Pseudomonadota were the dominant bacterial phyla across most dietary treatments, which is consistent with previous reports from lepidopteran insects (1, 6). However, the relative abundance patterns of these dominant groups differed among diets. The MS group showed increased representation of Actinomycetota-associated taxa, and LEfSe identified Actinomycetota-related features, including *Corynebacterium* associated taxa, in MS and Bacillota-related features in AD. Actinomycetota are widely distributed in soil, plants, and animal-associated environments, and some members possess diverse metabolic capacities, including secondary metabolite production and degradation of complex compounds (32). The presence of Actinomycetota-associated taxa in MS-fed larvae may therefore reflect differences in microbial input from food sources or selective conditions within the gut environment. However, taxonomic differences alone do not demonstrate specific ecological functions. For example, Enterococcus showed relatively higher abundance patterns in CA groups, and members of this genus have been reported to participate in insect–microbe interactions (33–36).

PICRUSt2 analysis revealed variation in the predicted functional potential of gut bacterial communities among dietary treatments. Metabolism-related pathways accounted for the largest proportion of the predicted functional profiles across all groups. The MS group showed relatively higher predicted abundances of amino acid metabolism and metabolism of terpenoids and polyketides, whereas the CA group showed higher predicted abundances of carbohydrate metabolism, nucleotide metabolism, and glycan biosynthesis and metabolism. Differences were also detected in several other predicted metabolic categories, including metabolism of cofactors and vitamins and xenobiotics biodegradation and metabolism. Comparable patterns have been reported in other lepidopteran insects. In *Grapholita molesta* reared on different host plants, predicted microbial functions differed among diets, including pathways related to amino acid transport and metabolism (37). In *S. frugiperda*, predicted pathways related to carbohydrate, nucleotide, lipid, xenobiotic, and amino acid metabolism differed between larvae fed an artificial diet and those fed maize leaves (38), while another study using multiple host plants also detected differences in predicted amino acid, energy, and cofactor and vitamin metabolism among dietary treatments (39). These observations indicate that diet-associated shifts in predicted microbial functional profiles are not unique to *L. sticticalis* and have also been observed in other polyphagous Lepidoptera. However, PICRUSt2 infers functional potential from 16S rRNA-based taxonomic profiles rather than directly measuring microbial genes, transcripts, metabolites, or enzyme activities (29). Previous benchmarking studies have further shown that the accuracy of 16S-based functional inference can vary substantially among sample types and functional categories, particularly in non-human microbial communities (40, 41). The nutritional composition and secondary metabolites of the experimental diets were not quantified in the present study. The observed pathway differences should thus be regarded as unvalidated differences in predicted microbial functional potential rather than evidence of microbial specialization, detoxification, nutrient provisioning, or adaptation to particular host plants. Direct functional assessment using metagenomic, transcriptomic, metabolomic, or experimental approaches will be required to determine their biological significance.

By comparing three botanically distinct host plants with an artificial diet under common larval rearing conditions, this study provides a comparative baseline for diet-associated gut bacterial variation in *L. sticticalis* and identifies bacterial taxa and predicted pathways that merit targeted investigation. Because food-associated microorganisms were not profiled and PICRUSt2 predictions were not experimentally validated, the mechanisms generating these patterns and their functional consequences remain to be determined. Future studies that combine diet-microbiota characterization with metagenomic, metabolomic will help distinguish microbial input from dietary filtering and test the roles of the candidate bacteria.

## 5 Conclusion

By comparing three botanically distinct host plants with an artificial diet under standardized larval rearing conditions, this study provides a comparative framework for characterizing diet-associated variation in the gut bacterial communities of *L. sticticalis*. The observed differences in bacterial diversity, community composition, differential taxa, and predicted functional profiles indicate that contrasting diets are associated with distinct microbiome patterns in this polyphagous insect. The higher bacterial diversity and increased representation of Actinomycetota associated taxa in the MS group, together with treatment-associated differences in predicted functional potential, identify microbial features that warrant further investigation and extend current knowledge of diet microbiota associations in *L. sticticalis*. Interpretation of these patterns should remain within the limits of the present experimental design because the study was based on three pooled biological replicates per treatment, while plant-surface microbiota and plant chemical composition were not directly characterized. PICRUSt2 derived functional profiles also represent predictions rather than experimentally validated microbial functions. The present results therefore support diet-associated differences in gut bacterial community structure and predicted functional potential, but do not establish specific mechanisms of microbial specialization or host adaptation. Future studies integrating plant-associated microbiota, plant chemistry, metagenomics, metabolomics, and host performance measurements will help determine the functional significance of the microbial patterns identified here.

## Data Availability

The original contributions presented in the study are publicly available. This data can be found here: NCBI Sequence Read Archive (SRA) under BioProject accession PRJNA1527117.

## References

[1] Engel P Moran NA . The gut microbiota of insects—diversity in structure and function. FEMS Microbiol Rev. (2013) 37:699–735. doi: 10.1111/1574-6976.12025 23692388

[2] Douglas AE . Multiorganismal insects: diversity and function of resident microorganisms. Annu Rev Entomol. (2015) 60:17–34. doi: 10.1146/annurev-ento-010814-020822 25341109 PMC4465791

[3] Engel P Martinson VG Moran NA . Functional diversity within the simple gut microbiota of the honey bee. Proc Natl Acad Sci USA. (2012) 109:11002–7. doi: 10.1073/pnas.1202970109 22711827 PMC3390884

[4] Yun JH Roh SW Whon TW Jung MJ Kim MS Park DS . Insect gut bacterial diversity determined by environmental habitat, diet, developmental stage, and phylogeny of host. Appl Environ Microbiol. (2014) 80:5254–64. doi: 10.1128/AEM.01226-14 24928884 PMC4136111

[5] Mason CJ . Complex relationships at the intersection of insect gut microbiomes and plant defenses. J Chem Ecol. (2020) 46:793–807. doi: 10.1007/s10886-020-01187-1 32537721

[6] Hammer TJ Janzen DH Hallwachs W Jaffe SP Fierer N . Caterpillars lack a resident gut microbiome. Proc Natl Acad Sci USA. (2017) 114:9641–6. doi: 10.1073/pnas.1707186114 28830993 PMC5594680

[7] Huang KG Wang J Huang JH Zhang SK Vogler AP Liu QQ . Host phylogeny and diet shape gut microbial communities within bamboo-feeding insects. Front Microbiol. (2021) 12:633075. doi: 10.3389/fmicb.2021.633075 34239504 PMC8260032

[8] Yan H Wang ED Wei GS Zhang B Xu XN . Both host and diet shape bacterial communities of predatory mites. Insect Sci. (2024) 31:551–61. doi: 10.1111/1744-7917.13253 37469127

[9] Shang LH Cai XY Li XJ Wang YZ Wang JD Hou YM . Role of gut bacteria in enhancing host adaptation of *Tuta absoluta* to different host plants. Insects. (2024) 15:795. doi: 10.3390/insects15100795 39452371 PMC11508330

[10] Do TH Dao TK Nguyen HD Truong NH . Understanding the role of free-living bacteria in the gut of the lower termite *Coptotermes gestroi* based on metagenomic DNA analysis. Insects. (2023) 14:832. doi: 10.3390/insects14110832 37999031 PMC10671698

[11] Berasategui A Salem H Paetz C Santoro M Gershenzon J Kaltenpoth M . Gut microbiota of the pine weevil degrades conifer diterpenes and increases insect fitness. Mol Ecol. (2017) 26:4099–110. doi: 10.1111/mec.14186 28543918

[12] Jones AG Charles JM Gary WF Kelli H . Host plant and population source drive diversity of microbial gut communities in two polyphagous insects. Sci Rep. (2019) 1:2792. doi: 10.1038/s41598-019-39163-9 30808905 PMC6391413

[13] Mason CJ Rubert-Nason KF Lindroth RL Raffa KF . Aspen defense chemicals influence midgut bacterial community composition of gypsy moth. J Chem Ecol. (2015) 41:75–84. doi: 10.1007/s10886-014-0530-1 25475786

[14] Mason CJ Hoover K Felton GW . Effects of maize (Zea mays) genotypes and microbial sources in shaping fall armyworm (Spodoptera frugiperda) gut bacterial communities. Sci Rep. (2021) 11:4429. doi: 10.1038/s41598-021-83497-2 33627698 PMC7904771

[15] Mugo-Kamiri L Querejeta M Raymond B Herniou EA . The effect of diet composition on the diversity of active gut bacteria and on the growth of Spodoptera exigua (Lepidoptera: Noctuidae). J Insect Sci. (2024) 24:13. doi: 10.1093/jisesa/ieae031 38513149 PMC10956968

[16] Kruttika P Krushnamegh K Deepa A . Dietary and developmental shifts in butterfly-associated bacterial communities. R Soc Open Sci. (2018) 5:171559. doi: 10.1098/rsos.171559 29892359 PMC5990769

[17] Qadri M Short S Gast K Hernandez J Wong ACN . Microbiome innovation in agriculture: development of microbial-based tools for insect pest management. Front Sustain Food Syst. (2020) 4:547751. doi: 10.3389/fsufs.2020.547751

[18] Huang SZ Zhang L Xie DJ Tang JH Jiang YY Mijidsuren B . Transboundary migration of *Loxostege sticticalis* (Lepidoptera: Crambidae) among China, Russia and Mongolia. Pest Manag Sci. (2024) 80:4650–64. doi: 10.1002/ps.8183 38775404

[19] Wen M Li E Chen Q Kang H Zhang S Li K . A herbivore-induced plant volatile of the host plant acts as a collective foraging signal to the larvae of the meadow moth, *Loxostege sticticalis* (Lepidoptera: Pyralidae). J Insect Physiol. (2019) 118:103941. doi: 10.1016/j.jinsphys.2019.103941 31499032

[20] Ji XW Liu YX Ding TB Chen JL Tan XL . Development and reproduction of *Loxostege sticticalis* fed with artificial diet. Plant Prot. (2025) 51:48–55. doi: 10.16688/j.zwbh.2024604

[21] Wang JY Chong HN Li D Cui SW Song YN He JY . Comparison of distinct gut bacterial communities in different stages of prediapause and nondiapause larvae in *Loxostege sticticalis*. Front Microbiol. (2024) 15:1469140. doi: 10.3389/fmicb.2024.1469140 39469464 PMC11513625

[22] Dai DF Yang Y Yu JQ Dang TF Qin WJ Teng LS . Interactions between gastric microbiota and metabolites in gastric cancer. Cell Death Dis. (2021) 12:1104. doi: 10.1038/s41419-021-04396-y 34819503 PMC8613192

[23] Callahan BJ McMurdie PJ Rosen MJ Han AW Johnson AJA Holmes SP . DADA2: high-resolution sample inference from Illumina amplicon data. Nat Methods. (2016) 13:581–3. doi: 10.1038/nmeth.3869 27214047 PMC4927377

[24] Bolyen E Rideout JR Dillon MR Bokulich NA Abnet CC Al-Ghalith GA . Reproducible, interactive, scalable and extensible microbiome data science using QIIME 2. Nat Biotechnol. (2019) 37:852–7. doi: 10.1038/s41587-019-0209-9 31341288 PMC7015180

[25] Bokulich NA Kaehler BD Rideout JR Dillon M Bolyen E Knight R . Optimizing taxonomic classification of marker-gene amplicon sequences with QIIME 2's q2-feature-classifier plugin. Microbiome. (2018) 6:90. doi: 10.1186/s40168-018-0470-z 29773078 PMC5956843

[26] Anderson MJ . Permutation tests for univariate or multivariate analysis of variance and regression. Can J Fish AquatSci. (2001) 58:626–39. doi: 10.1139/f01-004

[27] Anderson MJ . Distance-based tests for homogeneity of multivariate dispersions. Biometrics. (2006) 62:245–53. doi: 10.1111/j.1541-0420.2005.00440.x 16542252

[28] Segata N Izard J Waldron L Gevers D Miropolsky L Garrett WS . Metagenomic biomarker discovery and explanation. Genome Biol. (2011) 12:R60. doi: 10.1186/gb-2011-12-6-r60 21702898 PMC3218848

[29] Douglas GM Maffei VJ Zaneveld JR Yurgel SN Brown JR Taylor CM . PICRUSt2 for prediction of metagenome functions. Nat Biotechnol. (2020) 38:685–8. doi: 10.1038/s41587-020-0548-6 32483366 PMC7365738

[30] Chen YQ Zhou HC Lai YS Chen Q Yu XQ Wang XY . Gut microbiota dysbiosis influences metabolic homeostasis in *Spodoptera frugiperda*. Front Microbiol. (2021) 12:727434. doi: 10.3389/fmicb.2021.727434 34659154 PMC8514726

[31] Zhang SK Song F Wang J Li XY Zhang YX Zhou WW . Gut microbiota facilitate adaptation of invasive moths to new host plants. ISME J. (2024) 18:wrae031. doi: 10.1093/ismejo/wrae031 38423525 PMC10980833

[32] Barka EA Vatsa P Sanchez L Gaveau-Vaillant N Jacquard C Klenk HP . Taxonomy, physiology, and natural products of Actinobacteria. Microbiol Mol Biol Rev. (2016) 80:1–43. doi: 10.1128/MMBR.00044-16 26609051 PMC4711186

[33] Savio C Mugo-Kamiri L Upfold JK . Bugs in bugs: the role of probiotics and prebiotics in maintenance of health in mass-reared insects. Insects. (2022) 13:376. doi: 10.3390/insects13040376 35447818 PMC9025317

[34] Dow JA . pH gradients in lepidopteran midgut. J Exp Biol. (1992) 172:355–75. doi: 10.1242/jeb.172.1.355 9874748

[35] Zhang XC Feng HH He JT Liang XL Zhang N Shao YQ . The gut commensal bacterium *Enterococcus faecalis* LX10 contributes to defending against *Nosema bombycis* infection in *Bombyx mori*. Pest Manag Sci. (2022) 78:2215–27. doi: 10.1002/ps.6846 35192238 PMC9314687

[36] Broderick NA Raffa KF Goodman RM Handelsman J . Census of the bacterial community of the gypsy moth larval midgut by using culturing and culture-independent methods. Appl Environ Microbiol. (2004) 70:293–300. doi: 10.1128/AEM.70.1.293-300.2004 14711655 PMC321235

[37] Yuan X Zhang X Liu X Dong Y Yan Z Lv D . Comparison of gut bacterial communities of *Grapholita molesta* (Lepidoptera: Tortricidae) reared on different host plants. Int J Mol Sci. (2021) 22:6843. doi: 10.3390/ijms22136843 34202141 PMC8268091

[38] Xu X De Mandal S Wu H Zhu S Kong J Lin S . Effect of diet on the midgut microbial composition and host immunity of the fall armyworm, Spodoptera frugiperda. Biology. (2022) 11:1602. doi: 10.3390/biology11111602 36358303 PMC9687563

[39] Han S Zhou Y Wang D Qin Q Song P He Y . Effect of different host plants on the diversity of gut bacterial communities of *Spodoptera frugiperda* (J. E. Smith, 1797). Insects. (2023) 14:264. doi: 10.3390/insects14030264 36975949 PMC10053068

[40] MatChado MS Rühlemann M Reitmeier S Kacprowski T Frost F Haller D . On the limits of 16S rRNA gene-based metagenome prediction and functional profiling. Microb Genom. (2024) 10:1203. doi: 10.1099/mgen.0.001203 38421266 PMC10926695

[41] Sun S Jones RB Fodor AA . Inference-based accuracy of metagenome prediction tools varies across sample types and functional categories. Microbiome. (2020) 8:46. doi: 10.1186/s40168-020-00815-y 32241293 PMC7118876

